# A multiscale model reveals how ERK/p38-regulated dormancy shapes tumor-immune dynamics and immunoediting outcomes

**DOI:** 10.3389/fimmu.2026.1861769

**Published:** 2026-07-03

**Authors:** Eti Nyamekeh Baffoe, Anass Bouchnita

**Affiliations:** 1 Department of Mathematical Sciences, University of Texas at El Paso, El Paso, TX, United States; 2 Data Science Program, University of Texas at El Paso, El Paso, TX, United States

**Keywords:** dormancy, mathematical modeling, multiscale models, phenotype-structured models, systems immunology

## Abstract

**Introduction:**

Immune responses in cancer arise from dynamic interactions across biological scales, linking intracellular signaling, cellular phenotypic plasticity, and tumor–immune dynamics. Cancer cell dormancy is increasingly recognized as a continuous and heterogeneous phenotype driven in part by ERK and p38 signaling, yet most existing models represent it as a binary state, limiting their ability to capture its regulatory role in immune-mediated tumor control.

**Methods:**

Here, we introduce a new ERK-p38-structured model of cancer-immune cells in which tumor cells are continuously stratified along an ERK/p38 phenotypic axis. This framework provides a systems description that preserves both computational efficiency and theoretical tractability. It links intracellular signaling to cellular behavior and population-level dynamics by describing phenotype-dependent proliferation, immune susceptibility, and stress responses driven by growth factors, immune pressure, stress, and therapy.

**Results:**

Using this model, we show that the distribution of tumor phenotypes governs the emergent regimes of tumor–immune dynamics, including elimination, equilibrium, and escape. The model further predicts that increased p38 activation in response to immune pressure promotes immune evasion, whereas stress-induced p38 signaling drives global tumor dormancy. Conversely, p38 inhibition shifts the phenotype distribution toward more proliferative states, which enhances tumor sensitivity to immune-mediated killing and therapeutic interventions. To provide mechanistic insight, we derive a reduced model and identify critical thresholds in the mean ERK/p38 phenotype that determine tumor fate.

**Discussion:**

Together, these results suggest that phenotypic plasticity is a key regulator of immunoediting and provide a quantitative multiscale framework linking intracellular signaling to immune-driven tumor dynamics, with implications for understanding immune evasion and improving therapeutic responses.

## 1 Introduction

Cancer recurrence and immune escape are major challenges in oncology, driven in part by tumor cells that persist under immune pressure in dormant or slowly proliferative states. These cells can evade cytotoxic T lymphocyte (CTL)-mediated killing and survive immunotherapy, while retaining the ability to resume proliferation and drive relapse (1–3). Emerging evidence indicates that dormancy is not a uniform state but a heterogeneous phenotype, with variability in antigen presentation, metabolic activity, and susceptibility to immune-mediated clearance (4, 5). This heterogeneity shapes tumor–immune interactions and supports a view of dormancy as a dynamic immunological phenotype central to immune escape and immunoediting (6).

At the intracellular level, tumor dormancy is regulated by the balance between mitogenic and stress-responsive signaling pathways. In particular, the antagonistic interaction between extracellular signal–regulated kinase (ERK) and p38 MAPK signaling governs cell-cycle progression, stress adaptation, and survival (7). High ERK activity promotes proliferation, whereas elevated p38 activity is associated with growth arrest and stress resistance (8). Importantly, this signaling balance operates along a continuum, giving rise to intermediate phenotypic states that may differentially modulate immune recognition and susceptibility to cytotoxic responses (9). While these pathways are well characterized at the single-cell level, their role in shaping population-level tumor–immune dynamics and immunoediting remains poorly understood.

Mathematical and computational models have played an important role in elucidating general principles of tumor–immune interactions, including the emergence of immunoediting regimes such as elimination, equilibrium, and escape (10). Models studying cancer-immune dynamics can be broadly classified into continuous (11–14), discrete (15–17) and hybrid multiscale frameworks (18–21). Across these three categories, dormancy is often modeled as a discrete state that cancer cells switch into when faced with stress or immune pressure (22). Transitions between dormant and active states is usually modeled deterministically through compartmental or structured population models, but they can also be represented stochastically using branching processes, Gillespie-type birth-death models, cellular automata, individual-based models, or agent-based tumor-immune frameworks. These stochastic approaches are especially useful for studying rare escape events, relapse from small residual populations, and cell-level variability (16, 23).

Early models of tumor–immune interactions included immune-induced dormancy and characterized parameters controlling the dormant fraction in tumor populations (24). Other works have demonstrated how cancer-immune interactions and the microenvironment features regulate the development of a tumor dormancy state, characterized by slow growth (25). Simulations using a continuous stochastic framework have revealed that a small fraction of dormant cells provoke resistance against chemotherapy (26). Another hybrid stochastic models demonstrated that small populations of dormant cells could influence the timing of relapse following chemotherapy (27). In a previous work, we have shown that the administration of epidermal growth factor (EGF) can awaken dormant cells and sensitize them to immune checkpoint blockade (21).

While subdividing the tumor into proliferative and dormant cell populations improves analytical and numerical tractability, it inherently limits the ability of these models to capture graded phenotypic variability, such as intermediate dormancy states and selection acting on continuous dormancy traits. To address these limitations, phenotype-structured models were introduced to represent the dynamic evolution of cellular attributes driven by mutations, competition for resources, T-cell heterogeneity, and selective pressures imposed by therapeutic interventions or immune responses (12, 28–32). Here, we propose to apply this approach, for the first time, to capture the heterogeneity of cellular dormancy, regulated by ERK- and p38-signaling, and how it shapes the population dynamics of cancer-immune interactions.

In this work, we develop a structured model of tumor–immune interactions in which cancer cells are continuously distributed along an ERK/p38 signaling axis that governs immune susceptibility. We use phenotype-structured modeling because it flexibly describes intermediate states between activation and dormancy and represents movement across these states as a continuous process, which is central to the question of this study. As a result, dormancy is represented as a graded and dynamic immunological phenotype, rather than a discrete state. Cells undergo transport along this axis in response to competing forces, including growth factor signaling, stress responses, immune-mediated killing, and therapy. This framework provides, for the first time, a mechanistic link between ERK/p38 intracellular signaling heterogeneity and population-level tumor-immune dynamics. As a result, it enables analysis of how immune pressure selects for phenotypic states associated with immune evasion or clearance.

After introducing the model, we compare our model outputs to published experimental data on anti-p38 treatment. We find that modulation of p38 signaling reshapes the phenotypic distribution of tumor cells and increases sensitivity to both chemotherapy and immune checkpoint blockade. Using this validated model, we further show that the relative strength of ERK- and p38-mediated responses gives rise to distinct dynamical regimes corresponding to immune-mediated elimination, dynamic equilibrium, and escape. We also find that increased p38 responsiveness to immune pressure promotes immune evasion, whereas strong stress-induced p38 activation can drive global tumor dormancy. These results suggest that signaling-dependent dormancy heterogeneity can influence immunoediting trajectories.

Finally, to facilitate biological interpretation, we derive a reduced description of the model using a moment-based approach. This yields explicit thresholds in the mean ERK/p38 phenotype that delineate regimes of immune-mediated tumor elimination, control, or escape. These results provide a mechanistic interpretation of how phenotypic heterogeneity in dormancy regulates immune surveillance and evasion. Overall, the model identifies dormancy as a key determinant of immunoediting dynamics and suggests that shifts in the ERK/p38 balance may help the immune response control tumor progression.

## 2 Material and methods

### 2.1 An ERK/p38-structured model of tumor-immune interactions

**Figure 1:**
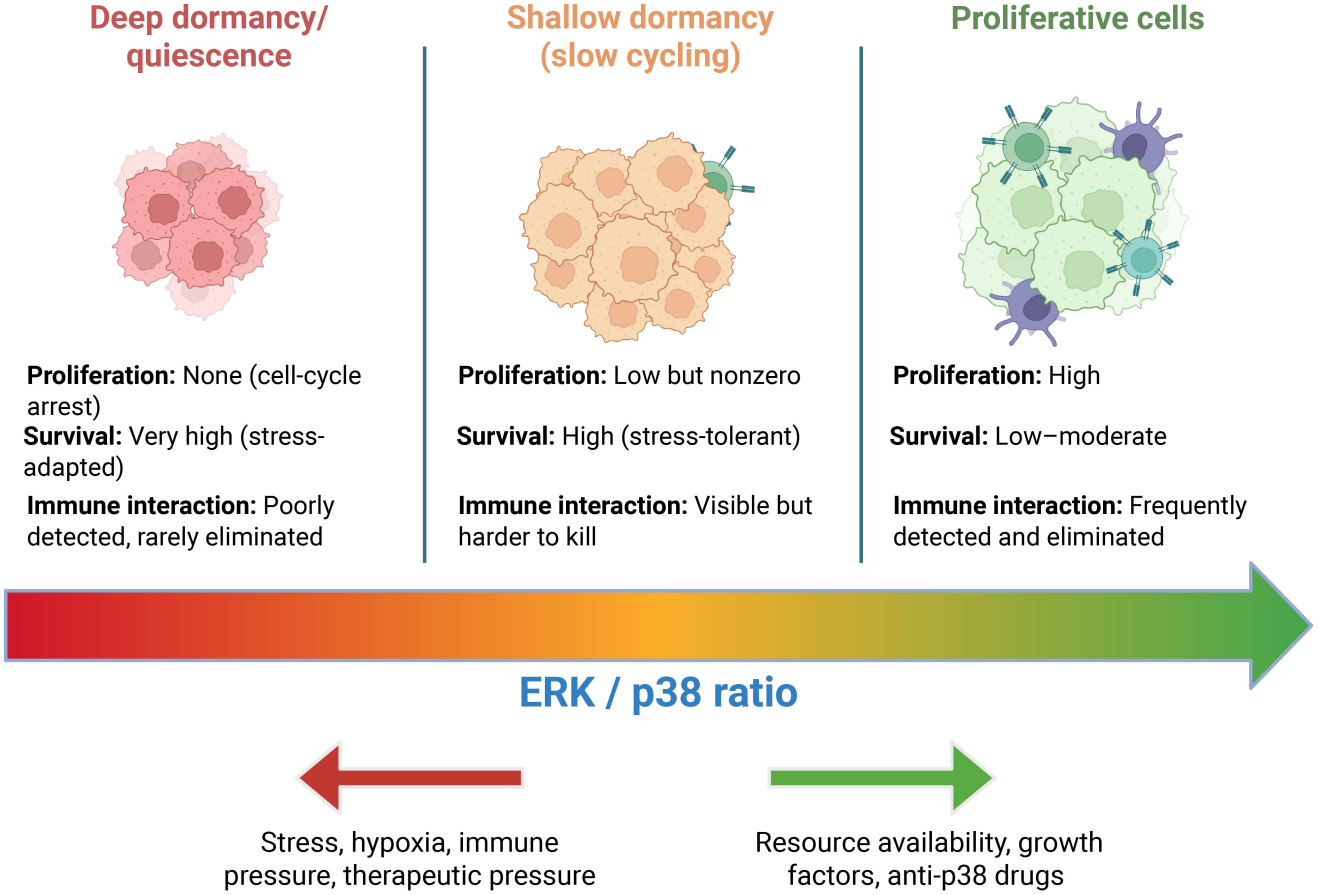
Schematic representation of the phenotypic continuum governed by the ERK/p38 index. The variable *x* ∈ [0,1] represents the relative dominance of ERK versus p38 signaling, with low values corresponding to dormancy and high values to proliferation. Transition forces along this axis arise from growth factors, immune pressure, stress, and therapy, and determine the evolution of the tumor phenotype distribution.

Here, we introduce a new model of cancer-immune interactions that explicitly accounts for phenotypic heterogeneity in cancer cell dormancy. We represent cancer cells along a continuous phenotypic axis *x* ∈ [0,1], which encodes the ERK/p38 dominance index. The value *x* = 0 corresponds to fully dormant cells, whereas *x* = 1 represents fully activated, proliferative cells. Cancer cells shift toward activation upon exposure to epidermal growth factor (EGF), which increases the ERK/p38 index. In contrast, they transition toward dormancy under EGF deprivation, immune pressure, or stress (**Figure 1**). Anti-p38 therapy promotes activation by inhibiting p38 signaling. Both proliferation and apoptosis depend on the phenotypic state: fully activated cells divide and die at maximal rates, while fully dormant cells neither proliferate nor undergo apoptosis. We further assume that proliferative cells induce stronger immune activation, whereas dormant cells exhibit enhanced immunosuppressive properties (33–35). The model tracks the dynamics of the following cell populations and molecular species:

- Cancer cells (*C*(*x,t*)), structured by phenotype *x*. Their proliferation and death rates depend on the ERK/p38 index.
- Cytotoxic T lymphocytes (*I*(*t*)), These cells are activated by cancer cells, eliminate tumor cells, and may become suppressed depending on tumor phenotype.
- Epidermal growth factor (*E*(*t*)), Cancer cells consume EGF, and it promotes their activation and proliferation.

The phenotypic trait *x* represents a normalized ERK-dominance index rather than a literal biochemical ratio, consistent with biological usage (36). Values close to 1 correspond to high ERK activity and low p38 activity, whereas values close to 0 indicate high p38 activity and low ERK activity. This formulation captures the relative dominance of these pathways on a phenomenological scale. At the population level, we model the evolution of the phenotype distribution through a transport equation in phenotype space:

(1) $$\frac{\partial C(x,t)}{\partial t}+\frac{\partial }{\partial x}(v(x,t)\;C(x,t))=P(x,t)-Q(x,t)-k_{8}I(t)xC(x,t),$$

where the second term on the left-hand side represents phenotypic transport along the ERK/p38 axis. This term follows from mass conservation over an infinitesimal interval [*x, x* + Δ*x*], where the flux is given by the product of the phenotypic velocity and the cell density. The phenotypic velocity *v*(*x,t*) describes the net effect of biological mechanisms driving transitions along the ERK/p38 axis:

(2) $$v(x,t)=\alpha E(t)(1-x)-\beta I(t)x-\gamma (E^{0}-E(t))x+\mu (1-x).$$

The first term in Equation 2 represents activation driven by EGF exposure, which increases ERK signaling (37). The second term captures the shift toward dormancy induced by immune pressure (38). The third term accounts for stress-induced dormancy under EGF deprivation (39). The final term represents activation due to anti-p38 therapy, with *µ* denoting treatment intensity (40). The multiplicative factors *x* and (1 − *x*) encode directional effects and ensure that the dynamics remain bounded within the interval [0,1]. Specifically, terms proportional to (1 − *x*) drive transitions toward activation (*x* → 1), while terms proportional to *x* drive transitions toward dormancy (*x* → 0). The first and second terms on the right-hand side of Equation 1 represent cell proliferation and apoptosis, respectively. We assume that daughter cells inherit the same ERK/p38 state and that this state regulates both division and death rates (41). These rates are maximal for fully activated cells (*x* = 1). Accordingly, we define


$$P(x,t)=k_{6}xC(x,t),\;\;Q(x,t)=k_{7}xC(x,t).$$

The total tumor burden (tumor volume) is defined as the integral of the phenotype distribution:

(3) $$\rho (t)=\int_{0}^{1}C(x,t)dx,$$

where the quantity introduced in Equation (3) describes the tumor volume compared with experiments. The final term in Equation 1 models immunemediated killing of cancer cells through the upregulation of apoptotic pathways (e.g., FASL and TRAIL) expressed by cytotoxic T lymphocytes (CTLs) (42). We assume this effect is strongest for highly proliferative cells (x ≈ 1), while dormant cells (x ≈ 0) can partially evade immune surveillance (33). We impose no-flux boundary conditions at x = 0 and x = 1:


v(x,t)C(x,t)=0 for x∈{0,1},

which ensure that cells remain within the admissible phenotypic domain [0,1]. As initial condition, we consider a population concentrated around intermediate dormancy levels:


$$C(x,0)=\{\begin{array}{ll} C_{0}, & \text{if}\;0.4<x<0.6,\\ 0, & \text{otherwise}, \end{array}$$

where *C*_0_ is chosen such that *ρ*(0) matches the initial tumor volume in experiments. We next describe the dynamics of cytotoxic T lymphocytes (CTLs) at the tumor site:

(4) $$\frac{dI(t)}{dt}=k_{9}(\int_{0}^{1}xC(x,t)dx)(I^{M}-I(t))-k_{10}(\int_{0}^{1}(1-x)C(x,t)dx)I(t)-k_{11}I(t).$$

The first term describes immune activation driven by highly proliferative tumor cells, which present stronger antigenic signals. The second term represents immune suppression mediated predominantly by dormant cells, which are associated with increased immune evasion mechanisms. Here, we consider that dormant cells suppress immunity not necessarily through the increased expression of immune checkpoints, but also through different mechanisms such as forming CTL-resistant niches and impairing local immune responses (43, 44). The third term accounts for the natural decay of CTLs. The initial condition is $I(0)=I^{0}.$

**Figure 2:**
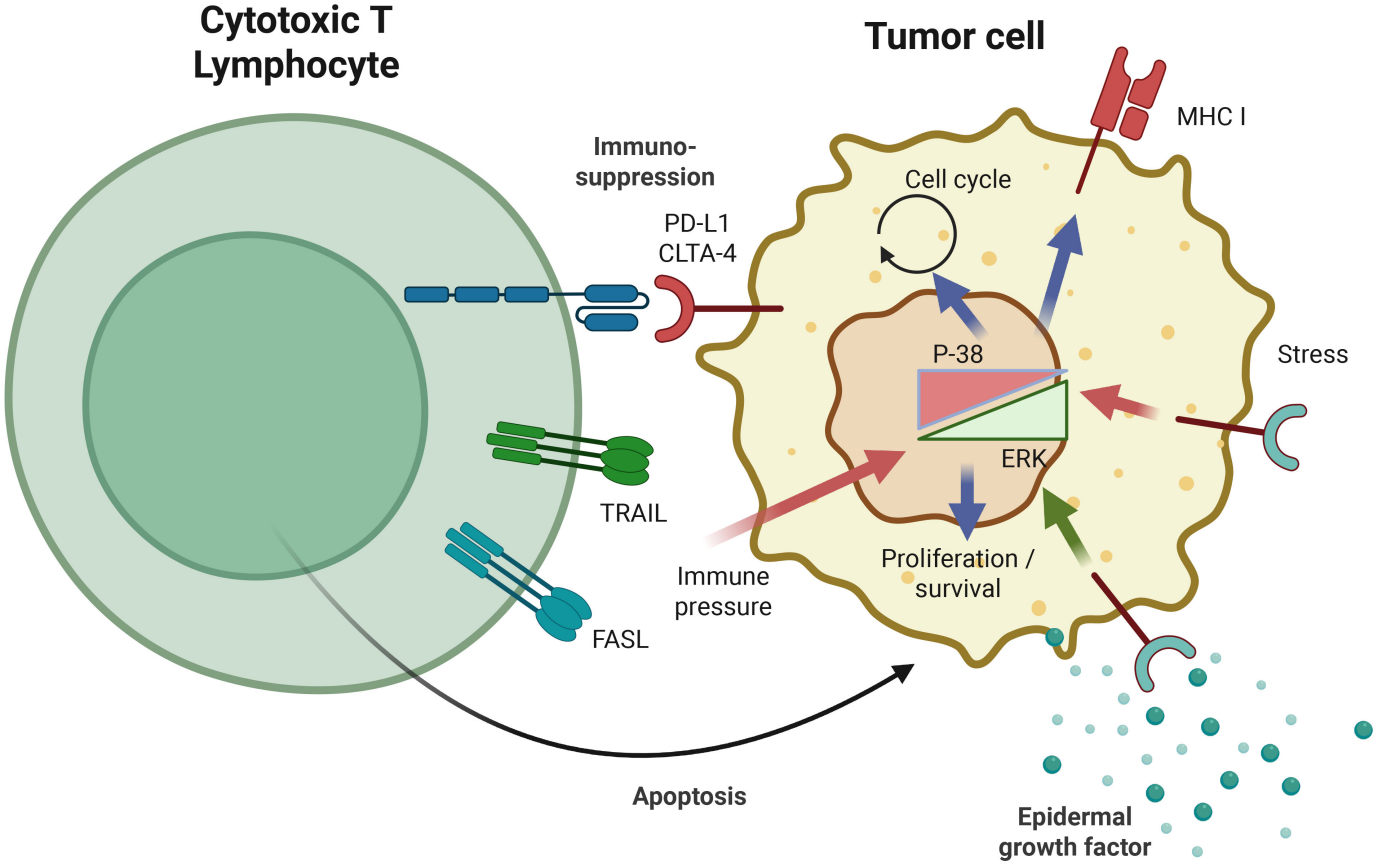
A schematic that illustrates the interactions between cancer cells and cytotoxic T lymphocytes (CTLs) captured by the model. CTLs induce apoptosis of cancer cells through the expression of FasL and TRAIL. They also impose immune pressure on tumor cells, which promotes p38 activation. Epidermal growth factor activates ERK, thereby modulating the ERK/p38 balance. This balance regulates cell cycle arrest, proliferation, and survival, as well as the expression of MHC-I and immune checkpoint molecules such as PD-L1 and CTLA-4. Created with Biorender.com.

Finally, we describe the concentration of epidermal growth factor (EGF) in the tissue:

(5) $$\frac{dE(t)}{dt}=k_{1}(E^{0}-E(t))-k_{2}E(t)\int_{0}^{1}xC(x,t)dx-k_{3}E(t),$$

where the first term represents influx toward a baseline level *E*^0^, the second term captures consumption by proliferative cancer cells, and the third term accounts for natural degradation. The initial condition is $E(0)=E^{0}$. EGF regulates phenotypic transitions along the ERK/p38 axis: high availability promotes activation, while depletion induces stress-driven shifts toward dormancy. The coupled interactions between tumor cells, CTLs, and EGF are summarized in **Figure 2**.

### 2.2 Parameter estimation

We collect population-level parameter values from the literature and from related modeling studies. The parameters governing ERK/p38 regulation are chosen phenomenologically and systematically explored in simulations, assuming that phenotypic changes occur on a timescale of minutes. Their baseline values are *α* = *γ* = 0.05 *ng*^−1^*.mL.day*^−1^, *β* = 1 × 10^−5^ *mm*^3^*.day*^−1^, and *µ* = 0 *day*^−1^. The baseline division rate and cancer elimination rate was slightly fitted within the physiological range to reproduce experiments (45). **Table 1** summarizes all fixed parameter values.

**Table 1:** Values of baseline model parameters with their biological interpretation and sources.

Parameter	Value	Description and reference
$E_{0}$	1 $ng.mL^{-1}$	EGF physiological concentration (46)
$k_{1}$	0.2 $day^{-1}$	EGF influx rate (46)
$k_{2}$	$1\times 10^{-8}$ $mm^{-3}.day^{-1}$	EGF consumption rate (47)
$k_{3}$	0.25 $day^{-1}$	EGF decay rate (48)
$C_{0}$	2.45 $mm^{3}$	Initial cancer volume for x∈[0.4,0.6] (45)
$k_{6}$	1.45 $day^{-1}$	Cancer baseline division rate (49)
$k_{7}$	1 $day^{-1}$	Cancer baseline apoptosis rate (49)
$k_{8}$	$10^{-5}$ $mm^{3}.day^{-1}$	Baseline elimination rate by CTLs (49)
α	0.05 $ng^{-1}.mL.day^{-1}$	ERK/p38 ratio increase rate in response to EGF (varied)
β	$1\times 10^{-5}$ $mm^{3}.day^{-1}$	ERK/p38 ratio decrease rate in response to immune pressure (varied)
γ	0.05 $ng^{-1}.mL.day^{-1}$	ERK/p38 ratio decrease rate in response to EGF scarcity (varied)
μ	0 $day^{-1}$	ERK/p38 ratio decrease rate in response to anti-p38 treatment (varied)
$k_{8}$	$10^{-5}$ $mm^{3}.day^{-1}$	Baseline elimination rate by CTLs (49)
$I^{0}$	$4\times 10^{3}$ mm−3	Initial CTL density (45)
$I^{M}$	$1\times 10^{7}$ mm−3	Maximum CTL density (50)
$k_{9}$	$2\times 10^{-8}$ $mm^{-3}.day^{-1}$	Baseline CTL activation rate (51)
$k_{10}$	$1\times 10^{-6}$ $mm^{-3}.day^{-1}$	Baseline CTL immunosuppression rate (49)
$k_{11}$	0.2 $day^{-1}$	CTL apoptosis rate (49)

### 2.3 Computer implementation

We solve the cancer cell equation (Equation 1) using a finite difference scheme. We discretize the domain *X* = [0,1] into 40 grid points, corresponding to a spatial step size of *dx* = 0.025, as determined by a consistency analysis. We set the simulation time step to *dt* = 0.00025 day. We implement the transport terms using an upwind scheme. The phenotype-structured equation was solved using an explicit finite-difference scheme on the normalized ERK/p38 domain *x* ∈ [0,1]. The domain was discretized using a uniform grid with spacing $dx$., and time was advanced with step size $dt$. The transport term was implemented with an upwind discretization, with the direction of differencing determined by the sign of each phenotypic drift contribution. Terms that drive cells toward higher ERK/p38 values were discretized with backward differences, whereas terms that drive cells toward lower ERK/p38 values were discretized with forward differences. This choice is consistent with the direction of phenotypic transport and reduces nonphysical numerical oscillations.

**Figure 3:**
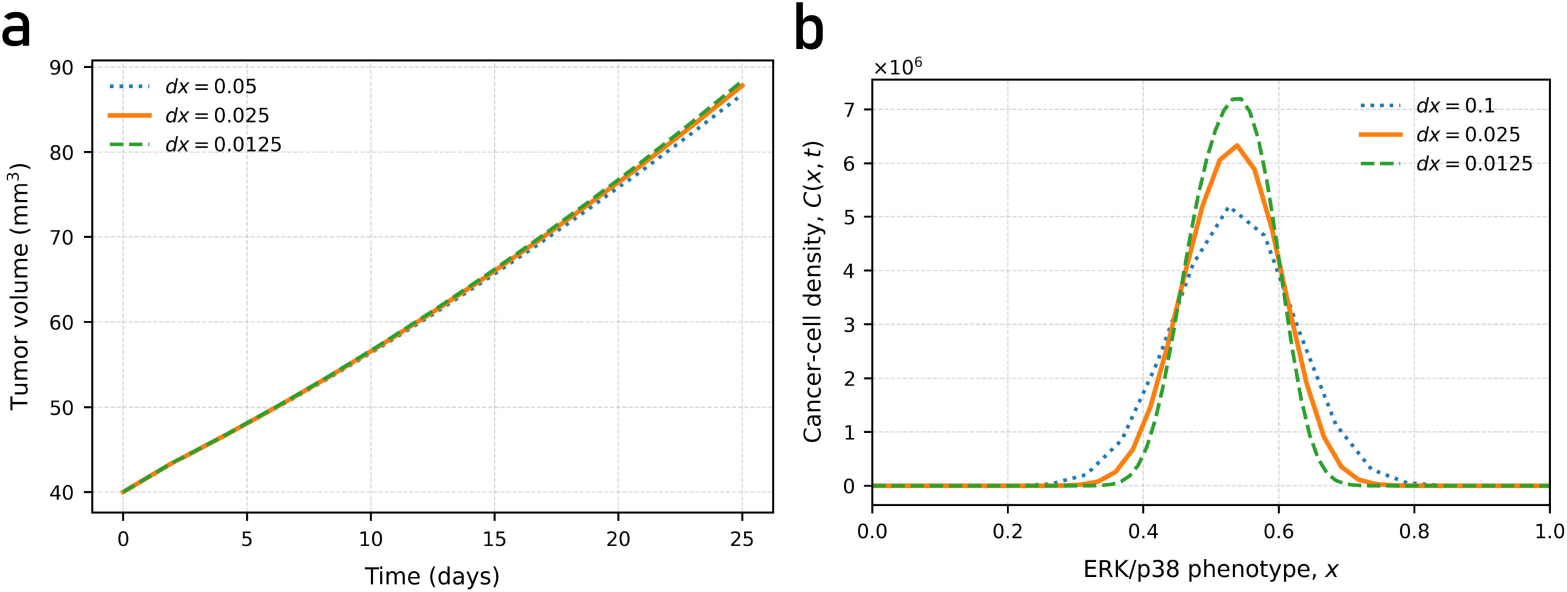
Grid-consistency analysis for the phenotype-structured numerical scheme. **(a)** Total tumor volume trajectories obtained with *dx* = 0.05, *dx* = 0.025, and *dx* = 0.0125 are nearly indistinguishable, indicating that the integrated tumor burden is insensitive to phenotype-grid refinement. **(b)** Phenotype-density profiles *C*(*x,t*) show small grid-dependent differences near the peak of the distribution, reflecting the local resolution of a narrow transported density. These differences do not materially affect the total tumor burden, *ρ*(*t*), which is the observable quantity used for calibration and comparison with experimental tumor-volume data.

At each time step, the EGF concentration and CTL density were first updated using an explicit Euler method. The cancer-cell density was then updated by combining the upwind transport contributions with 182 local proliferation, apoptosis, and CTL-mediated killing terms. In discrete form, the update has the structure


$$C_{j}^{n+1}=C_{j}^{n}-\text{Δ}tD_{x}^{\text{up}}(v_{j}^{n}C_{j}^{n})+\text{Δ}t[k_{6}x_{j}C_{j}^{n}-k_{7}x_{j}C_{j}^{n}-k_{8}I^{n}x_{j}C_{j}^{n}],$$

where $C_{j}^{n}$ denotes the cancer-cell density at phenotype grid point $x_{j}$ and time $t_{n}$ and $D_{x}^{\text{up}}$ denotes the upwind approximation of the phenotypic flux derivative. The integral terms appearing in the EGF and CTL equations were approximated by standard quadrature over the phenotype grid. The explicit scheme was used with a small time step to satisfy the transport Courant–Friedrichs–Lewy condition,


$$\text{Δ}t\;\frac{\underset{x,t}{\max}|v(x,t)|}{\Delta x}<1,$$

which ensures that phenotypic mass does not move by more than one grid cell during a single time step. We have added a grid-consistency analysis to justify the numerical discretization (**Figure 3**). The analysis compares simulations obtained with three phenotype-grid resolutions, *dx* = 0.05, *dx* = 0.025, and *dx* = 0.0125. The total tumor-volume trajectories are nearly indistinguishable across the three grids, indicating that the macroscopic quantity used for model calibration and biological interpretation is grid independent. Small grid-dependent differences remain in the pointwise phenotype-density profile *C*(*x,t*), particularly near the peak of the distribution. These differences reflect the local resolution of a narrow transported density and do not materially affect the integrated tumor burden, which is the observable quantity compared with experimental tumor-volume data. Thus, the discretization preserves the population-level dynamics relevant to the conclusions, while the remaining density-level differences are limited to the fine structure of the phenotype distribution.

The equations for immune cells (Equation 4) and EGF (Equation 5) are solved using an explicit Euler method. The code was implemented using a Jupyter notebook. Simulations corresponding to 25 days of tumor growth take approximately 30 seconds on a workstation equipped with AMD Ryzen Threadripper and 64 GB of RAM. The code is publicly available at https://github.com/MPS7/cancer_immune_ERK_p38.

## 3 Results

### 3.1 Model validation *in vivo* experiments for anti-p38 pre-clinical treatments

We begin our analysis by comparing the validation of the model against *in vivo* experiments for anti-p38 therapy (45). The role of PSKH1, an autophosphorylating protein serine kinase, in osteosarcoma (OS) progression was investigated using a combination of *in vitro* and *in vivo* experiments. Human OS cell lines were analyzed using Cell Counting Kit-8 assays, colony formation assays, wound healing assays, and Transwell migration and invasion assays to assess proliferation, motility, and invasive potential. PSKH1 expression levels were measured in OS tissues and compared with adjacent non-malignant tissues.

At the molecular level, PSKH1 was shown to upregulate phosphorylated p38. Pharmacological inhibition of p38 using SB203580 attenuated the tumor-promoting effects of PSKH1, indicating the involvement of the p38 MAPK pathway. *In vivo*, PSKH1 knockdown suppressed tumor growth and metastasis.

**Figure 4:**
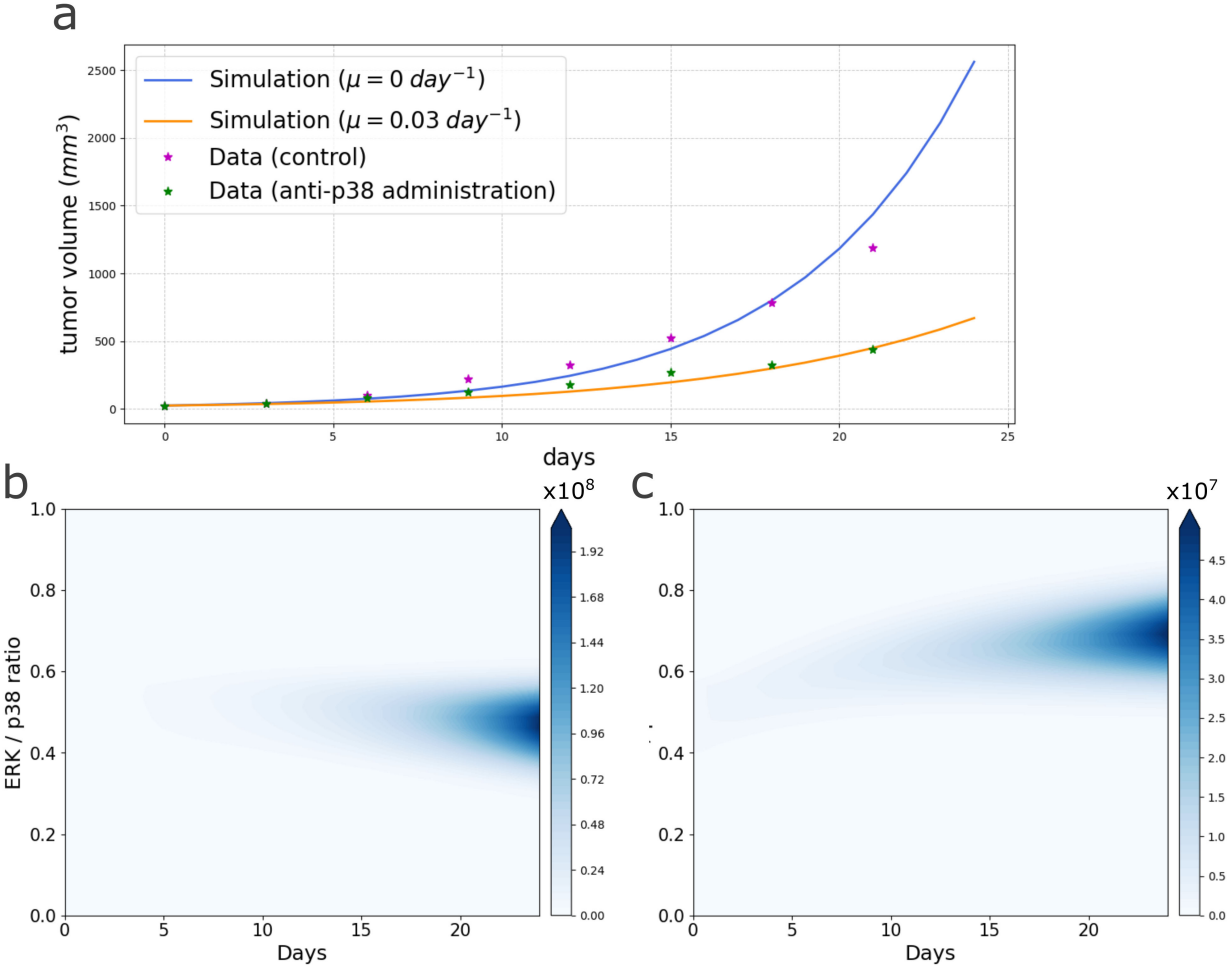
Validation of the mathematical model using *in vivo* experiments of tumor growth that uses anti-p38 to reduce cell proliferation (45). **(a)** A comparison between the experimental data (dots) and the fitted tumor growth simulations (curves). **(b)** The changes phenotype of cells over time in the control experiments. **(c)** The evolution of the cell phenotype in the case of anti-p38 administration.

We use these experimental results to validate our model. First, we manually fit the model to the vehicle condition by estimating the initial cancer cell density *C*_0_, the baseline proliferation rate (*k*_6_), and the baseline rate of CTL-mediated elimination (*k*_7_), restricting all parameters to their physiological ranges. We then increase the rate of ERK/p38 upregulation induced by anti-p38 treatment (*µ*) so that the simulated cancer cell population matches the experimental data (**Figure 4a**). The fitted value for the treatment effect is *µ* = 0.03 *day*^−1^. To further examine the effect of p38 inhibition on tumor growth, we analyze the evolution of the cancer cell phenotype along the ERK/p38 axis. In the absence of treatment, cells remain in a moderately proliferative, shallow dormancy state during the first 20 days. They subsequently drift toward deeper dormancy, driven by EGF scarcity (**Figure 4b**). Treatment alters this trajectory by shifting cells toward more proliferative states, as shown in **Figure 4c**. This shift increases their susceptibility to immune detection and CTL-mediated elimination. As a result, the total cancer cell population decreases.

To further assess the robustness and generalizability of the model, we validated it against an independent set of experiments testing the ULTR-p38 inhibitor 1639 in colorectal cancer (CRC) (52). These experiments provide a relevant validation setting because 1639 produces sustained p38*α* inhibition, suppresses downstream p38 signaling, and induces strong antitumor effects in CRC models. We kept all baseline parameters fixed and increased the maximum immune-cell density to *I^M^* = 3 × 10^7^ *mm*^−3^ to reproduce the carrier control curve. We then fitted only the anti-p38 treatment parameter, obtaining *µ* = 0.015 *day*^−1^ for the 1639-treated condition. Agreement between simulated and experimental tumor-volume trajectories under carrier and 1639 treatment provides an additional validation of the model’s ability to capture p38-regulated tumor dynamics beyond the original calibration dataset.

**Figure 5:**
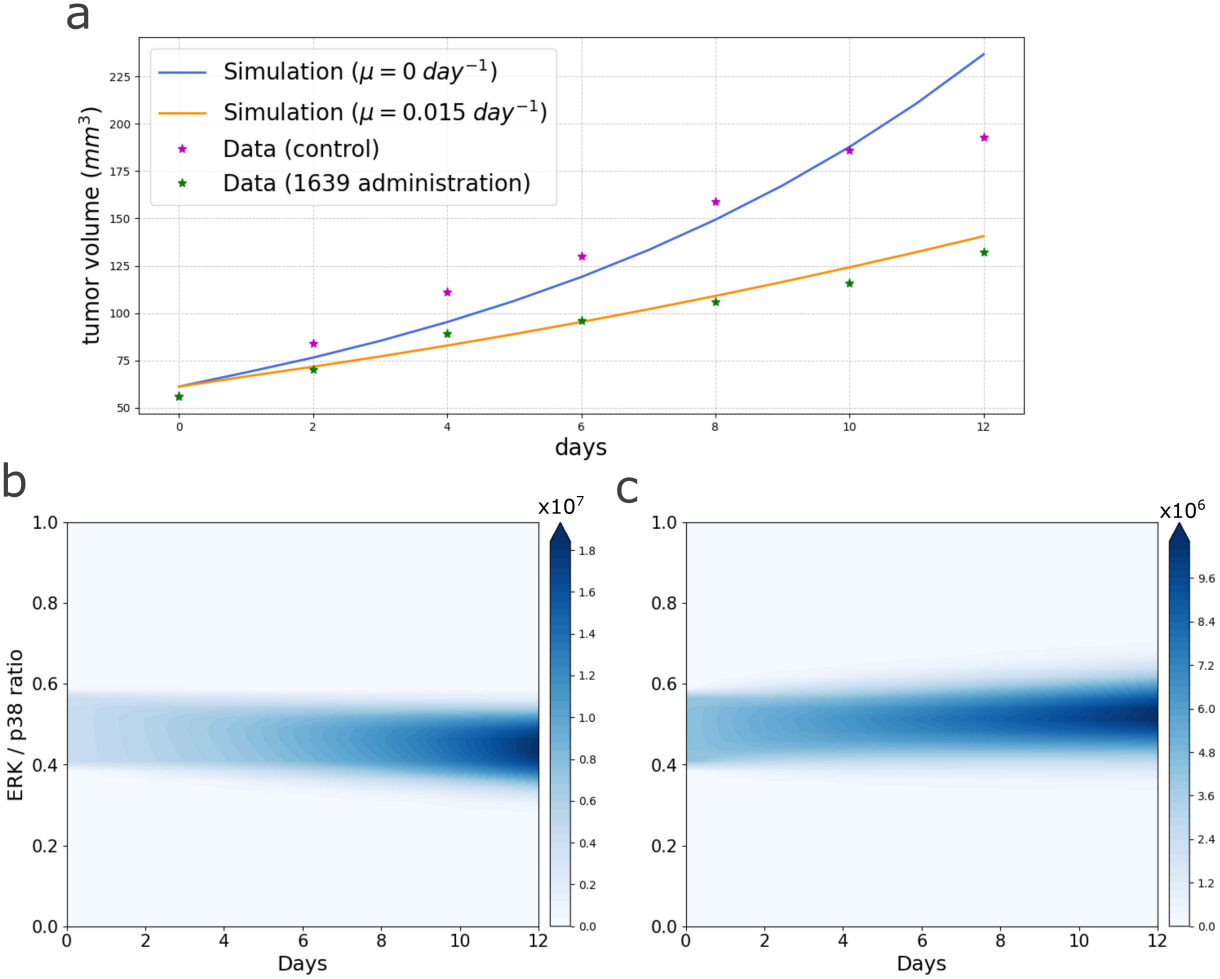
Second validation of the model using colorectal cancer experiments with the ULTR-p38 inhibitor 1639 (52). **(a)** Tumor-volume trajectories under carrier control and 1639 administration. At day 12, 1639 reduced tumor volume by approximately 32% relative to the carrier group. **(b, c)** Simulated phenotype distributions under carrier and 1639 treatment, respectively. The 1639-treated condition was reproduced by fitting the anti-p38 treatment parameter to *µ* = 0.015 day^−1^, while all other parameters were kept fixed except for the maximum immune-cell density, which was set to *I^M^* = 3 × 10^7^ cell.mm^−3^ to match the control trajectory.

The CRC validation experiment lasted for 12 days and showed a clear reduction in tumor growth under 1639 administration (**Figure 5a**). At day 12, the carrier group reached approximately 193 mm^3^, whereas the 1639-treated group reached approximately 132 mm^3^. This corresponds to an approximate 32% reduction in tumor volume. The model reproduced this treatment effect using *µ* = 0.015 day^−1^ and captured the slower tumor growth observed under p38 inhibition. The phenotype distributions further indicate that anti-p38 treatment shifts the tumor population toward higher ERK/p38 states (**Figures 5b,c**), which is consistent with the modeled mechanism of p38-regulated dormancy and treatment-induced phenotypic redistribution.

**Table 2** summarizes the calibration settings and goodness-of-fit metrics for the two independent validation experiments. The PSKH1 osteosarcoma dataset was fitted using an initial tumor volume of *C*_0_ = 20 mm^3^ and a maximum immune-cell density of *I^M^* = 1 × 10^7^ mm^−3^. For the CRC validation experiment with the 1639 p38 inhibitor, the initial tumor volume was set to *C*_0_ = 56 mm^3^, while *I^M^* was increased to 3×10^7^ mm^−3^ to match the carrier control trajectory. The lower mean squared error (MSE) values obtained for the CRC dataset indicate that the model reproduced both the control and anti-p38 treatment curves with high accuracy, providing additional support for the robustness of the model across distinct tumor contexts.

**Table 2:** Calibration settings and goodness-of-fit metrics for the two validation experiments. MSE stands for the mean squared error.

Experiment	PSKH1 osteosarcoma	1639 CRC
*C*_0_ (mm^3^)	20	56
*I^M^* (mm−3)	1 × 10^7^	3 × 10^7^
MSE control	5358.60	350.23
MSE anti-p38	2265.99	31.40
Combined MSE	3812.30	190.82

### 3.2 The inhibition of p38 promotes sensitivity to chemotherapy and immune checkpoint blockade

Following model validation, we investigate how p38 inhibition interacts with mechanisms that downregulate proliferation or reduce tumor immunosuppression. p38 MAPK is a central regulator of cellular stress responses, inflammation, and cell cycle arrest. Inhibition of p38 signaling can shift tumor cells toward a more proliferative and metabolically active state, thereby increasing their susceptibility to cytotoxic agents.

**Figure 6:**
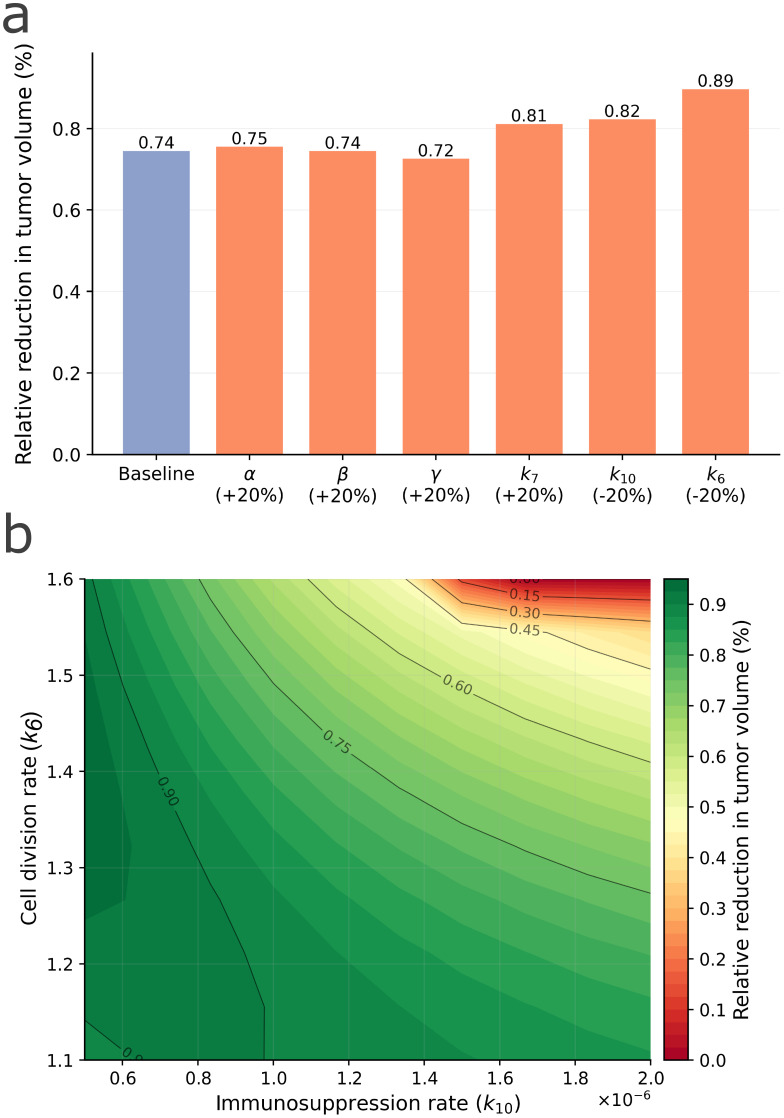
**(a)** Effect of small parameter perturbations on the reduction in tumor volume induced by anti-p38 treatment (*µ* = 0.03 day^−1^). **(b)** Tumor volume reduction under anti-p38 treatment (*µ* = 0.03 *day*^−1^) for simulations with varied cell proliferation and immunosuppression rates.

To assess robustness, we performed a local sensitivity analysis around the calibrated baseline parameter set. Parameters selected phenomenologically or modulated by treatments were perturbed one at a time, while all other parameters were kept fixed. We quantified the impact of each perturbation on final tumor burden, treatment-induced tumor-volume reduction, and qualitative tumor-immune outcome. This analysis was designed to determine whether the main conclusions depend on fine tuning of individual parameters or persist across a range of plausible values. Specifically, we used numerical simulations to evaluate the effect of a p38 inhibitor (*µ* = 0.03 *day*^−1^) in combination with changes in other model parameters (**Figure 6a**). Under baseline conditions, anti-p38 treatment reduced the tumor volume at day 25 by 74%. Increasing the cellular response to EGF further enhanced this reduction to 75%, whereas increasing the response to stress attenuated it to 72%. Modulating the response of the ERK/p38 axis to immune pressure did not significantly affect the tumor volume reduction. A marked reduction in tumor volume was achieved by modifying parameters that govern cancer-immune interactions. Specifically, increasing the CTL-mediated tumor elimination rate (*k*_7_) enhanced the tumor volume reduction to 81%, while decreasing the immunosuppression rate (*k*_10_) increased the reduction to 82%. Reducing the baseline cancer cell division rate (*k*_6_) further amplified the reduction to 89%. Together, these results indicate that decreasing immunosuppression and proliferation—clinically achievable through immune checkpoint blockade or chemotherapy-potentiates the therapeutic effect of p38 inhibition.

We further examine the effect of anti-p38 therapy while varying the cell division rate (*k*_6_) and the immunosuppression rate (*k*_10_) over wider ranges (**Figure 6b**). The resulting tumor volume reduction spans from approximately 10% for aggressive tumors with high immunosuppression to more than 90% for tumors with reduced immunosuppression and moderate proliferation. These sensitivity analysis results highlight the potential of combining p38 inhibitors with chemotherapy and immune checkpoint blockade.

### 3.3 The balance between the ERK and p38 responses to EGF availability and stress determines immunoediting outcomes

Cancer immunoediting describes the dynamic interplay between tumor cells and the immune system, encompassing three phases: elimination, equilibrium, and escape (53). During this process, immune pressure selects for tumor cell phenotypes with enhanced survival and immune evasion capacities. The balance between ERK and p38 MAPK signaling plays a central role in regulating these phenotypic states. ERK activity promotes proliferation, survival, and immune visibility, whereas p38 activation is associated with stress responses, quiescence, and therapy resistance (41). Shifts in the ERK/p38 balance therefore influence not only tumor growth dynamics but also immune recognition, dormancy, and escape, positioning this axis as a key regulator of immunoediting outcomes.

**Figure 7:**
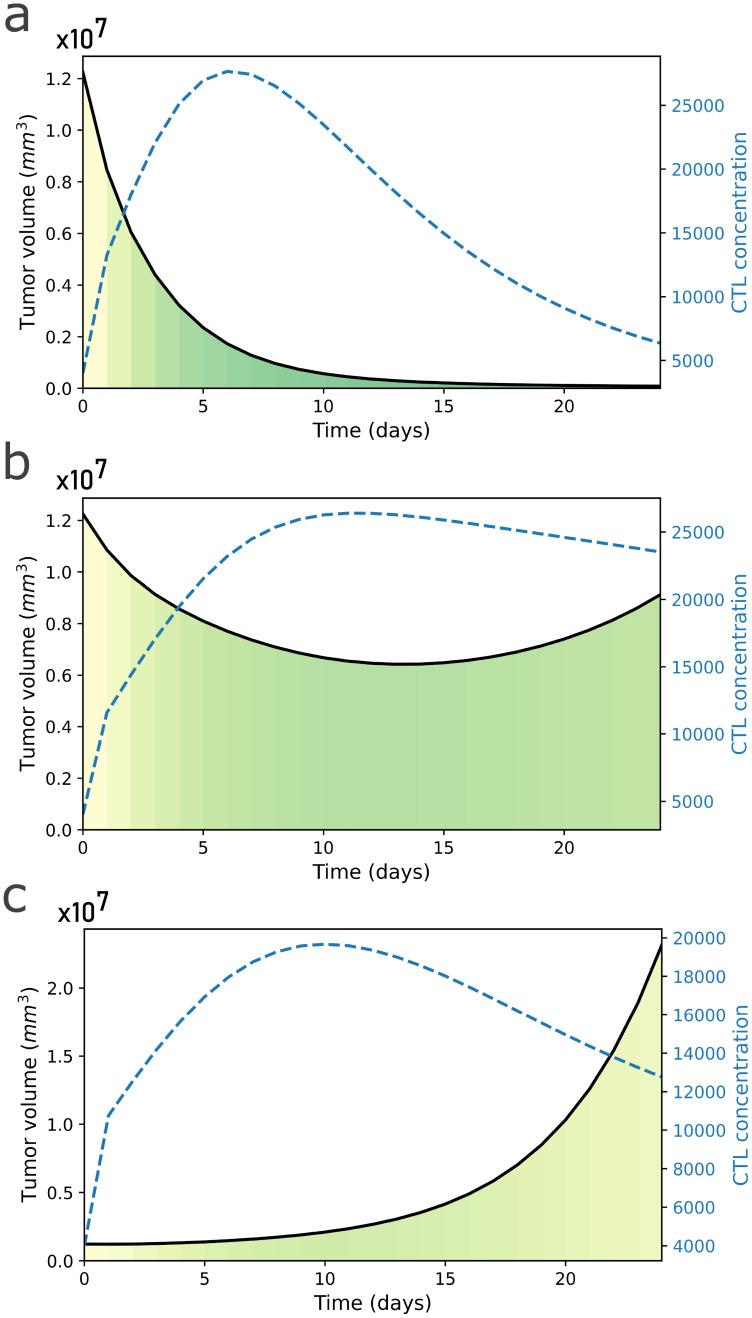
Three immunoediting regimes emerge as the relative cellular responses to EGF and stress are varied. The color gradient under the curve of tumor volume represent the mean ERK/p38 ratio, with red corresponding to 0 and green to 1. **(a)** Tumor elimination occurs when the ERK response to EGF strongly dominates the p38-mediated stress response (*α* = 30*γ*). **(b)** Tumor-immune equilibrium arises at intermediate dominance of ERK signaling (*α* = 17*γ*). **(c)** Tumor escape is observed when the ERK response is only moderately stronger than the p38 response (*α* = 11*γ*).

To examine how intracellular ERK and p38 regulation shapes tumor–immune dynamics, we vary the parameters *α* and *γ*, which control the response of the ERK/p38 index to EGF availability and stress, respectively. When the response to EGF is much stronger than the response to stress (*α* = 30*γ*), cancer cells shift toward highly proliferative states. These cells exhibit lower immunosuppression and higher susceptibility to CTL-mediated killing, leading to tumor elimination (**Figure 7a**). We then reduce the response to EGF to a moderate level (*α* = 17*γ*). In this case, the tumor volume initially decreases and then slowly increases, while the CTL population rises and subsequently declines (**Figure 7b**). This regime corresponds to a tumor-immune equilibrium state.

When the ERK response to EGF is only moderately stronger than the p38 response to stress (*α* = 11*γ*), cancer cells remain moderately proliferative while exhibiting strong immunosuppressive properties. This enables them to evade CTL-mediated elimination, leading to tumor escape and sustained growth. At later stages, the tumor further suppresses CTLs, which accelerates its expansion (**Figure 7c**). The outcomes of the three regimes are summarized in **Table 3**.

**Table 3:** Effect of increasing *α* on tumor-immune dynamics outcomes, measured by the tumor volume, the CTL density, and the mean ERK/p38 ratio on day 25.

Increase in *α*	Tumor volume	CTL density	Mean ERK/p38 ratio
30×	0 *mm*^3^	6,350 *mm*^−3^	0.92
17×	9.12 *mm*^3^	23,520 *mm*^−3^	0.71
11×	23,161 *mm*^3^	12,759 *mm*^−3^	0.56

### 3.4 Enhanced p38 response to immune surveillance promotes immune evasion by reducing immune activation

**Figure 8:**
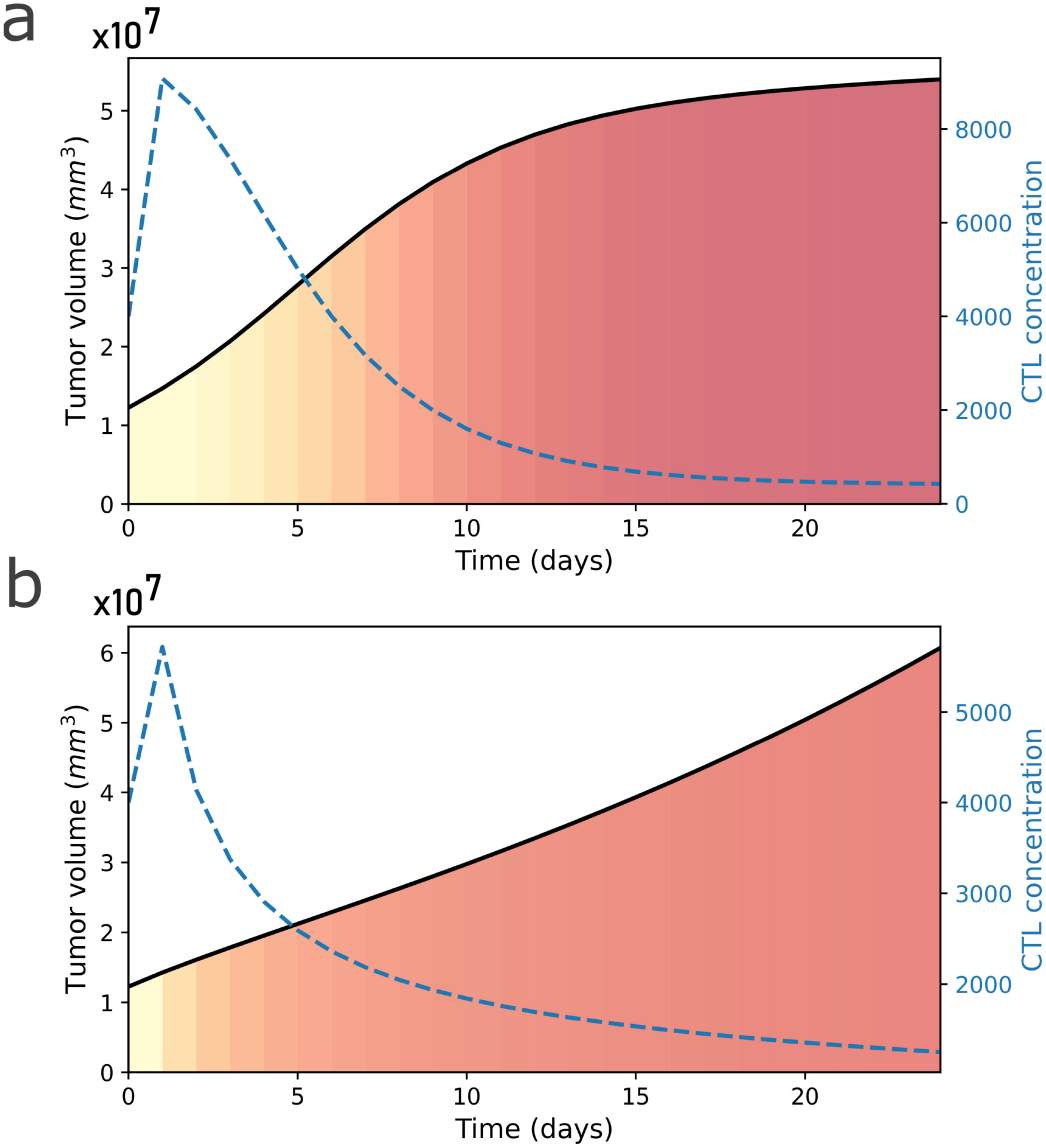
Tumor–immune interaction regimes when p38 signaling dominates over ERK. The color gradient along the curves indicates the mean ERK/p38 level, with red representing full dormancy (*x* = 0) and green full activation (*x* = 1). **(a)** Tumor dormancy regime, characterized by growth arrest, obtained when the ERK response to EGF is negligible compared to the p38 response to stress. **(b)** Dormant escape regime, characterized by a stronger p38 response to immune pressure.

After examining regimes in which the ERK response to EGF dominates the p38 stress response, we next consider the opposite scenario, where p38 signaling prevails. Specifically, we set the p38 response to stress to be five times stronger than the ERK response to EGF (*γ* = 5*α*). In this regime, the tumor initially escapes immune surveillance and grows slowly. As it becomes sufficiently large, the population shifts toward predominantly dormant cells, causing growth to arrest. As cancer cells become dormant, the activation of the immune response decreases which makes the tumor become undetectable to immune surveillance. This behavior corresponds to a tumor dormancy regime (**Figure 8a**). We next consider the case in which the p38 response to stress matches the ERK response to EGF, while the p38 response to immune pressure is fifty times larger than its baseline value (*β* = 50 × 10^−5^ mm^3^ day^−1^). In this regime, tumor cells detect immune surveillance earlier and transition toward dormant states. This shift enables them to evade CTL-mediated elimination and reduces their activation level. As a consequence, the tumor elicits only a low-amplitude immune response and continues to grow approximately linearly (**Figure 8b**). This behavior corresponds to the dormant escape regime. **Table 4** summarizes the results obtained for the two regimes where p38 signaling dominates over ERK.

**Table 4:** Effect of increasing *α* on tumor-immune dynamics outcomes in the case of p38-signaling dominated regimes.

p38 response	Tumor volume	CTL density	Mean ERK/p38 ratio
*γ* = 5*α*	53.98 *mm*^3^	427 *mm*^−3^	0.04
*β* = 50 × 10^−5^ mm^3^ day^−1^	60.71 *mm*^3^	1,205 *mm*^−3^	0.11

The outcomes are measured by the tumor volume, the CTL density, and the mean ERK/p38 ratio on day 25.

### 3.5 The mean ERK/p38 ratio that enables tumor elimination

To gain further insight into the model dynamics, we apply a moment reduction approach to derive a simplified ODE system that preserves the qualitative behavior of the full model (54). We focus on the first moment, defined as the mean ERK/p38 ratio in the population:


$$\bar{x}\left(t\right)\;=\;\frac{\int_{0}^{1}x\;C\left(x,\;t\right)\;dx}{\int_{0}^{1}C\left(x,\;t\right)\;dx}.$$

The reduced system tracks the variables *e*(*t*), *c*(*t*), *i*(*t*), and $\bar{x}(t)$, corresponding to EGF, cancer cells, CTLs, and the mean ERK/p38 ratio, respectively. Assuming that there are no saturation for the activation of CTLs to simplify the analysis, the reduced system is given by

(6) $$\frac{de}{dt}=k_{1}(e^{0}-e)-k_{2}e\bar{x}c-k_{3}e,$$

(7) $$\frac{dc}{dt}=k_{6}\bar{x}c-k_{7}i\bar{x}c-k_{8}\bar{x}c,$$

(8) $$\frac{di}{dt}=k_{9}\bar{x}c-k_{10}(1-\bar{x})ci-k_{11}i,$$

(9) $$\frac{d\bar{x}}{dt}=\alpha e(1-\bar{x})-\beta i\bar{x}-\gamma (E^{0}-e)\bar{x}-\mu \bar{x}.$$

Since the EGF dynamics regulate the evolution of the mean ERK/p38 ratio, we further simplify the system (Equations 6–9) by treating $\bar{x}$ as an independent control variable. Under this assumption, the dynamics can of the system can be reduced to (7) and (8). The tumor nullcline is given by

(10) $$c=0\;\mathrm{or}\;i=i_{\text{crit}}=\frac{k_{6}-k_{8}}{k_{7}},$$

where Equation 10 provides estimate for $i_{crit}$, the CTL level required is the CTL level required to offset net tumor growth (assuming k6 > k8). The CTL nullcline is

(11) $$i=0\;\mathrm{or}\;i=\frac{k_{9}\bar{x}c}{k_{10}(1-\bar{x})c+k_{11}}.$$

The nontrivial branch in (11) is increasing in *c* and saturates at

(12) $$i_{\max\;\;}(\bar{x})=\underset{c\to \infty }{\lim}\frac{k_{9}\bar{x}\;c}{k_{10}(1-\bar{x})c+k_{11}}=\frac{k_{9}\bar{x}}{k_{10}(1-\bar{x})}.$$

The system admits the tumor-free equilibrium (*c*^∗^*,i*^∗^) = (0,0). A coexistence equilibrium (*c*^∗^*,i*^∗^) with *c*^∗^ *>* 0 and *i*^∗^ *>* 0 exists when the nullclines (10)–(11) intersect for *c >* 0, yielding

(13) $$i^{*}=\frac{k_{6}-k_{8}}{k_{7}},\;\;c^{*}=\frac{(k_{6}-k_{8})k_{11}}{k_{7}k_{9}\bar{x}-(k_{6}-k_{8})k_{10}(1-\bar{x})}.$$

A necessary requirement for immune control is that CTLs can reach levels exceeding the critical killing threshold *i*_crit_ in (10). Since *i*(*t*) is bounded above by the saturation level $i_{\text{max }\;}(\bar{x})$ in (12), immune-mediated control is impossible if $i_{\text{max }\;}(\bar{x})\leq i_{\text{crit}}$. The inequality $i_{\text{max }\;}(\bar{x})>i_{\text{crit}}$ yields the threshold

(14) $$\bar{x}>{\bar{x}}^{*},\;\;{\bar{x}}^{*}=\frac{(k_{6}-k_{8})k_{10}}{k_{7}k_{9}+(k_{6}-k_{8})k_{10}}.$$

**Figure 9:**
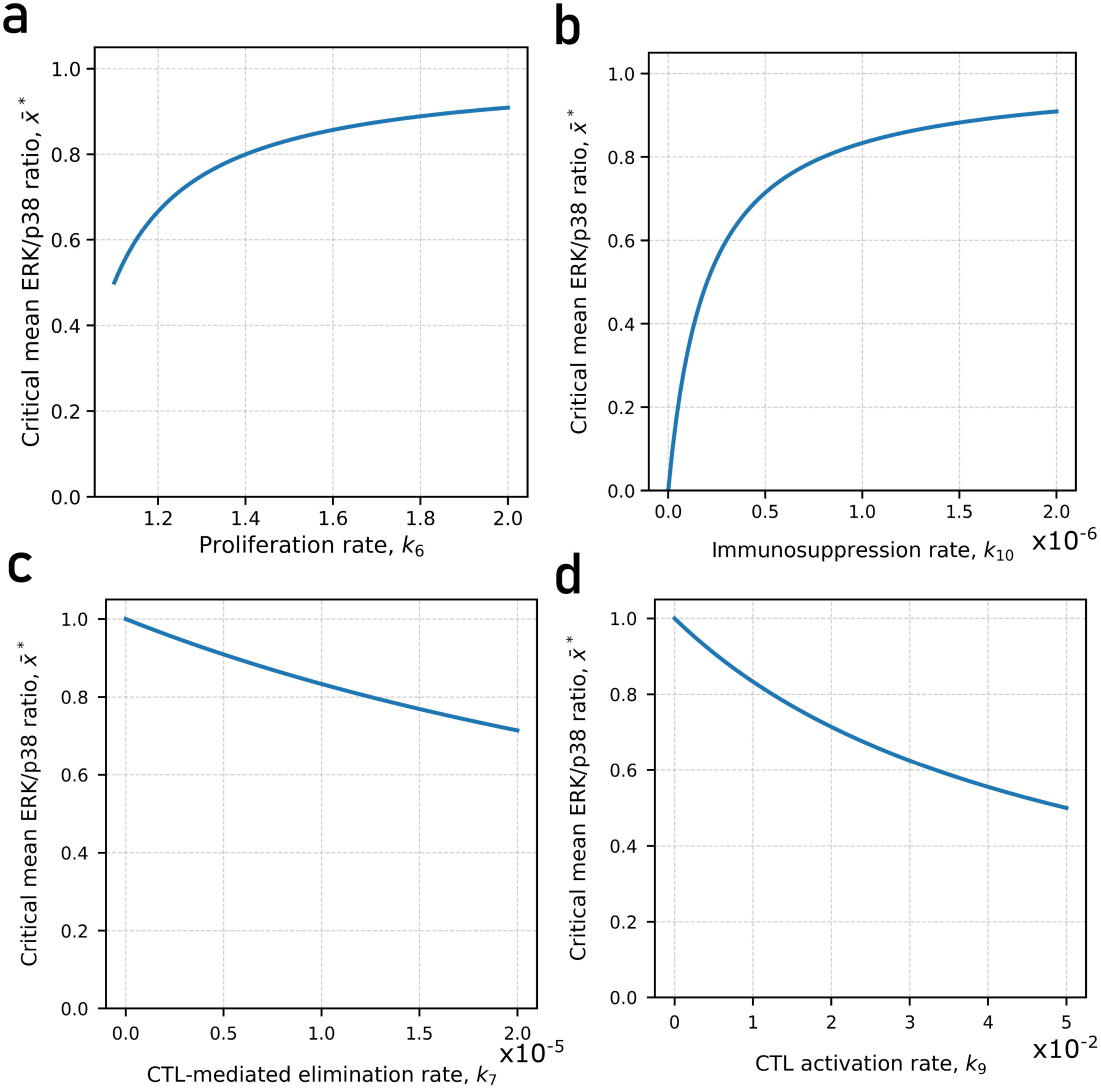
The mean ERK/p38 ratio required for tumor elimination as a function of the cancer proliferation rate **(a)**, immunosuppression rate **(b)**, CTL-mediated elimination rate **(c)**, and CTL activation rate **(d)**.

Equation 14 provides the condition for tumor elimination. If $\bar{x}<{\bar{x}}^{*}$, then $i_{\text{max }\;}(\bar{x})<i_{\text{crit}}$ and CTLs cannot reach the level required for net tumor decline; consequently, sustained tumor persistence/escape is expected in this reduced description. If $\bar{x}>{\bar{x}}^{*}$, then CTLs can exceed *i*_crit_, and trajectories may enter a regime where dc/dt<0 (immune dominance). In simulations and in the full phenotype-structured model (where $\bar{x}$ evolves dynamically), this condition corresponds to practical tumor elimination (i.e., *c*(*t*) driven below detection/extinction thresholds) because increased $\bar{x}$ both enhances immune activation (via $k_{9}\bar{x}c$) and weakens immunosuppression (via $k_{10}(1-\bar{x})ci$). The threshold value of the mean ERK/p38 ratio required for tumor elimination increases with higher cell division and immunosuppression rates (**Figures 9a, b**). In contrast, it decreases when CTL activation and elimination rates are reduced (**Figures 9c, d**).

## 4 Discussion

This work introduces a novel phenotype-structured model of tumor–immune interactions that integrates ERK/p38 regulatory dynamics within a computationally efficient and analytically tractable framework. By stratifying cancer cells along a continuous ERK/p38 axis, the model captures dynamic transitions between proliferative, adaptive, and dormant states, providing a unified representation of tumor phenotypic plasticity. The framework reproduces *in vivo* tumor growth dynamics under anti-p38 therapy (45) and predicts that p38 inhibition enhances sensitivity to chemotherapy and immune checkpoint blockade. Importantly, our results identify the ERK/p38 ratio as a central regulator of immunoediting, governing transitions between immune-mediated elimination, equilibrium, and escape. Beyond classical regimes, the model reveals additional interaction states emerging under dominant p38 signaling, corresponding to global tumor dormancy and dormant escape.

We speculate that the ability of the ERK/p38 coordinate to organize these regimes reflects the broad involvement of ERK and p38 MAPK pathways in biological processes that are central to tumor-immune dynamics, including cell-cycle progression, apoptosis, stress adaptation, metabolic regulation, therapy resistance, immunosuppressive signaling, and T-cell activation. Thus, the ERK/p38 axis provides a biologically motivated reduced coordinate that links intracellular signaling to phenotypic plasticity and immune susceptibility. Other pathways, including IFN-*γ*/JAK–STAT, NF-*κ*B, PI3K/AKT, and cytokine-mediated feedbacks, may quantitatively influence checkpoint expression, immune recognition, and tumor persistence. However, the purpose of the present model is to isolate the contribution of a well-supported ERK/p38 regulatory axis within a minimal and tractable tumor-immune framework. Additionally, the framework could be interpreted as generalizable at the level of biological structure, while quantitative predictions remain tumor-type dependent. The additional CRC validation provides support beyond the original osteosarcoma dataset, but cancer types may differ in growth kinetics, immune composition, stromal structure, and sensitivity to p38 inhibition. Therefore, the predicted thresholds and treatment effects should be interpreted as context-specific model outputs that require tumor-specific calibration and further validation.

Most existing tumor–immune models treat cancer cells as homogeneous populations or divide them into a small number of discrete phenotypic classes. While several studies have incorporated dormancy or quiescence, these states are typically modeled as binary compartments or externally imposed transitions. In contrast, our framework introduces a continuous, biologically grounded description of dormancy governed by the ERK/p38 signaling balance. This approach captures gradual, reversible phenotypic transitions between proliferative, stress-adapted, and dormant states. By explicitly coupling this intracellular signaling axis to immune activation, immunosuppression, and killing, our model identifies phenotypic plasticity rather than genetic change alone-as a central driver of immunoediting. To our knowledge, this is the first tumor-immune model that treats dormancy as a continuous, mechanistically interpretable trait directly regulating immune interactions.

The conclusions of our analysis are supported by several experimental and clinical studies. First, experimental evidence supports the ERK-p38 signaling balance as a phenotypic switch that can shape tumor-immune outcomes. In hepatocellular carcinoma cells, the EGFR–p38 MAPK axis was reported to upregulate PD-L1 and also affect HLA-I expression, directly connecting p38/ERK-related signaling to checkpoint-mediated immune evasion (55). Another experimental study showed that a high ERK/p38 ratio favors tumor growth, whereas a high p38/ERK ratio induces growth arrest (dormancy) *in vivo*, and that cells can acquire escape mechanisms from p38-driven growth inhibition, which agrees with our model predictions (36).

Second, several studies have shown that p38 inhibition can enhance the efficacy of both chemotherapy and immune checkpoint blockade (ICB) (56–58). However, other reports have highlighted potential safety concerns that may limit the clinical translation of these approaches, including hepatotoxicity and skin toxicity (59). These findings underscore the importance of interpreting our results as preclinical, testable hypotheses that require further validation in safety and efficacy studies.

Finally, experimental evidence supports the existence of tumor-immune interaction regimes characterized by full tumor dormancy. In the biological literature, tumor dormancy refers to a prolonged state of cell cycle arrest or to a balance between proliferation and death, resulting in no net tumor growth. This state may occur at the level of single quiescent cells (cellular dormancy) or as small tumor masses maintained under immune or microenvironmental control. Our model reproduces both of these regimes. Consistent with this behavior, mouse models have demonstrated that adaptive immune responses can maintain preformed cancer cells in a dormant state, leading to stable, non-expanding tumor masses (60).

Despite its ability to capture key features of immunoediting and dormancy, our model has several limitations which were introduced to reduce complexity and enable the interpretation of the results. First, the model does not explicitly resolve cytokines such as IL-2, IFN-*γ*, or TNF-*α*, although these mediators play important roles in T-cell activation, tumor killing, inflammation, and immune-mediated dormancy. Their effects are represented at an aggregate level through CTL activation, tumor-induced suppression, and CTL-mediated killing terms. This simplification allows us to reduce the complexity and improve the interpretability of the results. Second, the immune compartment is represented by a single effector CTL population. *In vivo*, tumor-immune interactions also involve NK cells, macrophages, dendritic cells, regulatory T cells, and additional stromal and inflammatory signals. These populations may amplify, suppress, or redirect the CTL response and could therefore modulate the quantitative thresholds predicted by the model. However, the role of CTLs remain more significant as their dynamics characterizes the adaptive nature of the immune response. This simplified structure preserves analytical tractability and allows the model to identify core mechanisms of dormancy-driven immunoediting. Future extensions can incorporate additional immune populations and cytokine feedbacks as coupled compartments or phenotype-dependent interaction terms within the same framework. Third, although ERK/p38 signaling has been linked to proliferation, stress responses, and dormancy, the regulation of immune checkpoints such as PD-L1 is known to involve additional pathways, including IFN-*γ*/JAK–STAT and NF-*κ*B signaling (61). Consequently, PD-L1 expression is not uniquely determined by the ERK/p38 balance in all biological contexts. A further limitation concerns parameter identifiability. Several population-level parameters were fixed or constrained using values from biological assays and published tumor-immune modeling studies. However, the ERK/p38 regulatory parameters were introduced as effective phenomenological quantities that summarize pathway activation, stress response, immune pressure, and antip38 treatment effects. Because the available validation data consist primarily of tumor-volume trajectories, these signaling parameters cannot be uniquely identified from the data alone. They should therefore be interpreted as effective parameters describing the relative strength and time scale of ERK/p38-mediated phenotypic transitions, rather than as direct biochemical rate constants.

The present framework should be interpreted as generalizable at the level of model structure, while quantitative predictions remain context dependent. The ERK/p38 axis is a biologically motivated reduced coordinate because ERK signaling is linked to mitogenic growth and cell-cycle progression, whereas p38 signaling is associated with stress responses, apoptosis, growth arrest, and dormancy (36). However, tumor types differ in growth kinetics, immune composition, cytokine signaling, stromal structure, and sensitivity to p38 inhibition. Thus, the additional colorectal cancer validation supports the broader applicability of the framework, but tumor-type-specific calibration and further validation remain necessary for quantitative prediction.

## 5 Conclusion

Overall, our analysis identifies the ERK/p38 balance as a biologically interpretable indicator of dormancy-associated tumor phenotype and immune susceptibility. This interpretation is consistent with experimental studies showing that ERK dominance promotes tumor growth, whereas p38 dominance is associated with stress adaptation, growth arrest, and dormancy (36). Within the present model, shifts in the ERK/p38 balance organize the transition between immune-mediated elimination, equilibrium, escape, global dormancy, and dormant escape. Thus, the ERK/p38 axis provides a compact and mechanistically grounded coordinate for linking intracellular signaling, phenotypic plasticity, and tumor-immune outcomes. The present framework establishes a scalable formalism for tumor-immune modeling which can support systematic integration of additional signaling pathways, cytokine-mediated feedbacks, and pharmacokinetic processes within a unified multiscale description.

## Data Availability

The original contributions presented in the study are included in the article. Further inquiries can be directed to the corresponding author.
